# Arterial Hypertonus, Sclerosis and Blood Pressure

**Published:** 1908-09

**Authors:** 


**Arterial Hypertonus, Sclerosis and Blood Pressure.** By William Russell, M.B. Pp. ix., 194. Edinburgh: William Green and Sons. 1907.

This is a very interesting book and well worth study, if we wish to understand what the pulse and the sphygmometer really indicate, whether we accept all the writer's conclusions or no. He argues that "thickened" or "hard arteries" are most frequently due to a hypertrophied muscular coat, and that this is often in a state of excessive contraction, sometimes even causing a feeling of rings like those in true atheroma. These "hypertonic" vessels readily relax and contract, and are perhaps more prone to do so than normal vessels.

Arterial contraction and relaxation, again, is largely produced by the direct action on the vessel walls of various substances in the blood, and not only by the vaso-motor nerves. This action affects not only the periphery, but the greater part of the vascular system. The brachial and radial vessels contract as well as the arterioles or capillaries. Contraction produces a rise of pressure, strictly speaking, above or behind the contraction, not necessarily in the contracted area. When we apply a sphygmometer to the brachial we find sometimes pressures of 150 to 250 mm. indicated, yet we know that in young and healthy males the pressure does not exceed 95 to 120, which under violent exertion can be raised to 140, the extra 20 representing the reserve power of the heart. Is it possible that the hearts of weak old people, then, can sustain a pressure of 200 to 250 mm. ?

Russell's reply is that these apparently enormous pressures are chiefly due to the resistance of the thickened vessel wall, and only in small part to the blood pressure. Still, sphygmometer readings are of value, for they show, by their variations from day to day, the varying condition of the patient and the effect of remedies; while as to normal vessels, he allows that their results have been confirmed by manometers in the living body.

He supports his argument by tests on a mechanical model of ingenious construction, but it is rather hard to see how he would account for the rise to 300 or 400 mm. which occurs in traumatic cerebral compression. Hypertrophy of the muscular coat cannot occur in an hour.

There is a useful chapter on the high tension produced by constipation, excess of proteid, and in some persons by alcohol; but he gives no proof that alcohol has any action except as a gastric irritant, and does not even refer to its dilator effects. We question whether the fashionable ascription of disease to intestinal toxins has much more proof than the animal spirits or miasma of older writers. Still, it is a working hypothesis.

He applies his theories with much success to the explanation of transient and recurrent cerebral paralyses, showing that many cases are probably due to hypertonic contraction or spasm of cerebral vessels. Though these vessels are not governed by the systemic vaso-motor centre, they may be acted on directly by toxins in the blood. Transient paralyses in uræmia are perhaps of this type, and the importance of distinguishing them from those due to hemorrhage or thrombosis is very great.

The whole book is most suggestive, though much of it is in direct opposition to accepted ideas, and much may ere long be shown to be incorrect.

